# Can myocardial susceptibility quantification be an imaging biomarker for cardiac amyloidosis?

**DOI:** 10.1007/s11604-021-01228-z

**Published:** 2021-11-29

**Authors:** Hidetaka Hayashi, Seitaro Oda, Masafumi Kidoh, Takeshi Nakaura, Kosuke Morita, Yasunori Nagayama, Tetsuya Yoneda, Seiji Takashio, Yohei Misumi, Mitsuharu Ueda, Kenichi Tsujita, Toshinori Hirai

**Affiliations:** 1 Department of Diagnostic Radiology, Faculty of Life Sciences, Kumamoto University, 1-1-1 Honjyo, Chuo-ku, Kumamoto, 860-8556 Japan; 2 Department of Central Radiology, Kumamoto University Hospital, Kumamoto, Japan; 3 Department of Medical Physics in Advanced Biomedical Sciences, Faculty of Life Sciences, Kumamoto University, Kumamoto, Japan; 4 Department of Cardiovascular Medicine, Faculty of Life Sciences, Kumamoto University, Kumamoto, Japan; 5 Department of Neurology, Faculty of Life Sciences, Kumamoto University, Kumamoto, Japan

**Keywords:** Cardiac amyloidosis, Imaging biomarker, Cardiac magnetic resonance imaging, Myocardial susceptibility

## Abstract

**Purpose:**

This study aimed to evaluate whether quantification of myocardial susceptibility by cardiac magnetic resonance imaging (CMR) can be an imaging biomarker for cardiac amyloidosis (CA).

**Materials and methods:**

Twenty-six patients with CA underwent CMR, including magnetic phase imaging with a 3.0-T magnetic resonance imaging scanner. Myocardial susceptibility was quantified as a phase shift slope value by magnetic phase analysis. Those values from patients with CA were compared with corresponding values from 18 controls and 15 healthy volunteers. A univariate logistic regression analysis was conducted to identify significant parameters related to CA.

**Results:**

The phase shift slope, a quantitative parameter of myocardial susceptibility, was significantly lower in the CA group compared with the control group and compared with healthy volunteers (*p* < 0.01). From a total of 17 tested variables, 6 were considered to be significant predictors of CA (*p* ≤ 0.05) during the univariate analysis. The phase shift slope yielded the best AUC of 0.89 (95% CI = 0.79–0.98) for the prediction of CA (*p* < 0.01). The phase shift slope was significantly correlated with the end-diastolic thickness of the interventricular septum (*r* =  − 0.39, *p* < 0.01) and posterior wall of the left ventricle (*r* =  − 0.35, *p* = 0.02).

**Conclusion:**

Myocardial susceptibility analysis by CMR helps in the diagnosis of patients with CA and can be a new quantitative imaging biomarker for CA.

## Introduction

Cardiac amyloidosis (CA) is caused by an accumulation of amyloid fibrils in myocardial interstitium, which induces a characteristic progressive and restrictive infiltrative cardiomyopathy. Cardiac involvement is the most significant predictor of poor prognosis in patients with systemic amyloidosis [1]. Amyloid fibrils may deposit within any region of the heart, including the myocardial interstitium, vessels, endocardium, valves, epicardium, and parietal pericardium. This deposition leads to progressive diastolic and systolic dysfunction, congestive heart failure, and conduction diseases, such as atrioventricular block, which may lead to faintness, syncope, sudden death, and occasional ischemia [2]. CA is generally considered a rare disease. However, hidden CA is increasingly recognized in patients with heart failure, especially those with preserved ejection fraction, who are also classified as diastolic heart failure. Although the true incidence of CA is probably underestimated, early diagnosis and intervention are critical in preventing rapid worsening of prognosis due to advancing organ dysfunction.

Cardiac magnetic resonance imaging (CMR, also known as cardiac MRI) using late gadolinium enhancement (LGE) imaging helps in the diagnosis of cardiac involvement in patients with systemic amyloidosis [3]. The assessment of LGE is important because it is strongly associated with clinical, morphological, functional, and biochemical markers that help evaluate patients with CA [4]. However, LGE is not easily quantitated and not reliable for objective evaluations of disease status. Myocardial T1 mapping, a novel CMR technique that enables noninvasive detection and quantification of myocardial damage, is becoming a popular method for the diagnosis of CA [5].

Recently, attention has been focused on performing more specific diagnosis by multiparametric analysis using a number of the nonspecific quantitative imaging biomarkers, including T1 mapping. Some previous studies have reported the potential value of multiparametric CMR analysis in identifying cardiac damage [6]. In the more sophisticated diagnosis of CA, it is desirable to find new quantitative imaging parameters with different mechanisms from existing ones for multiparametric analysis.

Magnetic susceptibility describes the extent to which a substance becomes magnetized when placed in an external magnetic field. Many biological tissues exhibit either positive or negative susceptibility and are termed paramagnetic or diamagnetic, respectively. Moreover, some of these tissues also have susceptibilities that are dependent on tissue orientation. Particularly, striated muscle tissue is known to exhibit anisotropic magnetic susceptibility. Although susceptibility-weighted imaging successfully demonstrated that field inhomogeneity induced by susceptibility could be used to infer the tissue property, the missing link between the observed phenomenon and underlying cause was not connected, and it does not provide quantitative measures of magnetic susceptibility. Local susceptibility directly or indirectly affects phase differences in magnetic resonance phase images. Therefore, it is possible to quantify local susceptibility based on phase differences. Quantitative susceptibility imaging (mapping) is a new MRI technique that aims to extract the spatial susceptibility distribution of tissues from the measured MRI phase or local field data [7]. To the best of our knowledge, there have been no attempts that have assessed the feasibility of myocardial susceptibility quantification for the diagnosis of CA.

This study aimed to evaluate whether the quantification of myocardial susceptibility by CMR can be an imaging biomarker for CA.

## Materials and methods

### Study population

This study was conducted in accordance with the principles outlined in the Declaration of Helsinki. The retrospective study was approved by our institutional review board, and patient informed consent was waived. Between July 2015 and July 2016, 26 patients with CA (17 men and 9 women, aged 61.5 ± 13.9 years (mean ± standard deviation); range, 28–86 years) and no contraindications for CMR were enrolled in this study. Eighteen patients had hereditary (variant) transthyretin amyloidosis, six had wild-type transthyretin amyloidosis, and two had immunoglobulin light-chain amyloidosis. CA was diagnosed based on clinical findings, amyloid deposition, and genetic testing. It was histologically confirmed by Congo red and immunohistochemical staining of bone marrow, soft tissue, fat, upper gastrointestinal tract, rectal, and liver specimens. Eighteen patients (10 men and 8 women, ages 59.0 ± 15.9 years, range 26–79 years) clinically referred for CMR had normal heart structure and function and were age- and sex matched to the CA group as control (control group). Clinical indication of CMR in patients assigned as controls was abnormal electrocardiogram or history of syncope. Fifteen healthy volunteers (15 men, age 35.5 ± 5.9 years, range 28–50 years) with no prior cardiac history or symptoms of cardiovascular disease or known cardiac risk factors, who had normal electrocardiographic findings, and who did not take any cardiovascular medications were also enrolled in this study. Informed consent was obtained from healthy volunteers before their participation in this study. Three patients with CA were excluded from the study because of the poor image quality.

### Echocardiography

All patients underwent standard echocardiographic studies on a commercial ultrasound machine (Vivid 7, GE Healthcare, Milwaukee, WI, USA) equipped with a 3.5-MHz transducer. The examinations were performed by experienced investigators. All measurements were performed in accordance with the current American Society of Echocardiography and European Association of Echocardiography guidelines [8, 9]. We measured left ventricular end-diastolic and end-systolic dimensions and end-diastolic thickness of the interventricular septum (IVSTd) and posterior wall of the left ventricle (PWTd) using the standard parasternal short-axis view with M-mode echocardiography. To measure the peak, early diastolic velocity at the tip of the mitral valve (*E* velocity), deceleration time of the mitral inflow, and mitral annular velocity at the septal annulus (*e*ʹ velocity), we used the standard apical four-chamber view.

### CMR protocol

All CMR studies were performed on a 3.0-T MRI scanner (Ingenia CX, Philips Healthcare, Best, The Netherlands). The patients were scanned in the supine position using a 16-channel phased-array coil. Images were acquired using T2-weighted black blood with the short tau inversion recovery. Cine imaging was obtained with a segmented steady-state free-precession sequence. LGE imaging was performed in short- and three long-axis views (four-, two-, and three-chamber views) with a mid-diastolic inversion-prepared two-dimensional (2D) gradient echo (GRE) sequence and three-dimensional (3D) phase-sensitive inversion recovery sequence. Data acquisition started 8 min after injection of 0.2 mmol/kg of a gadobutrol (Gadovist, Bayer Yakuhin, Ltd., Osaka, Japan).

**Fig. 1:**
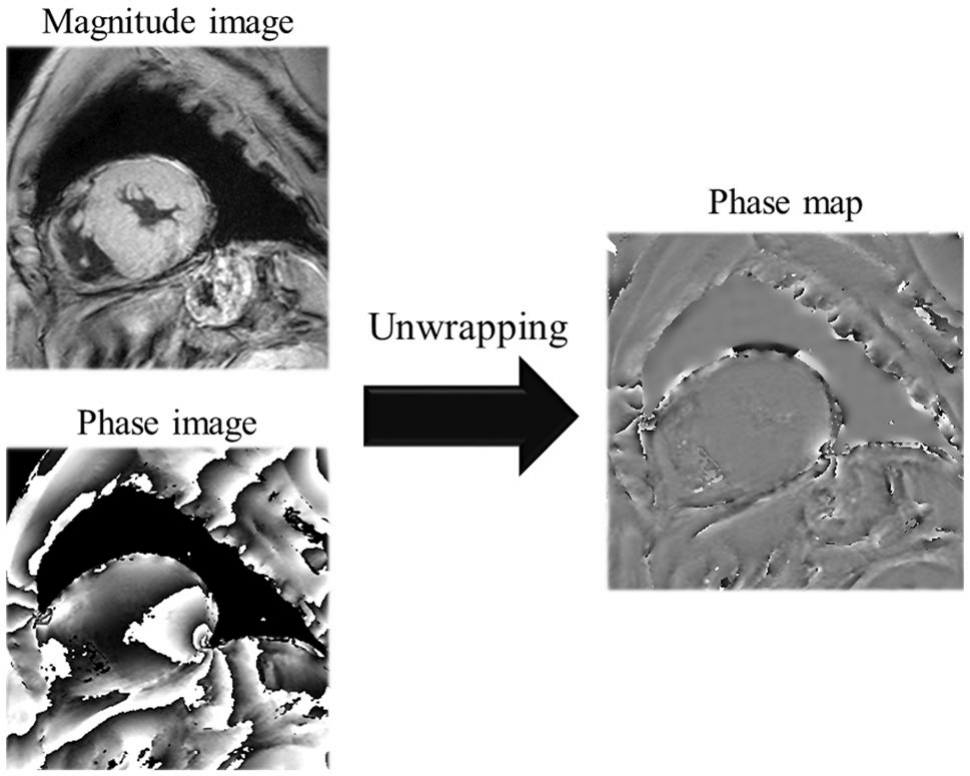
Creation of phase map. Both magnitude and phase images were generated from each echo. All phase data were processed using an integrated phase unwrapping algorithm to create the phase map

Magnetic phase imaging was performed in the short-axis image plane of the left ventricle using a multi-echo 2D spoiled GRE sequence with electrocardiography-triggered breath holding and black-blood inversion recovery pre-pulse. The sequence acquired data at four different echo times (3.9, 7.8, 11.8, and 15.7 ms). The imaging parameters included: repetition time = 18 ms, echo time = 3.8–15.2 ms (four echoes), flip angle = 20°, field of view = 200 × 200 mm, SENSE facto*r* = 2.0, acquisition matrix size = 1.25 × 1.56 mm, reconstruction matrix size = 0.63 × 0.63 mm, slice thickness = 7.0 mm, bandwidth = 289.4 Hz per pixel, scan duration = 14.8 s and black-blood pulse. Cardiac triggering was set for mid-diastole to reduce motion artifact. Both magnitude and phase images were generated from each echo. All phase data were processed using an integrated phase unwrapping algorithm that created phase maps (Fig. 1).

### Image analysis

**Fig. 2:**
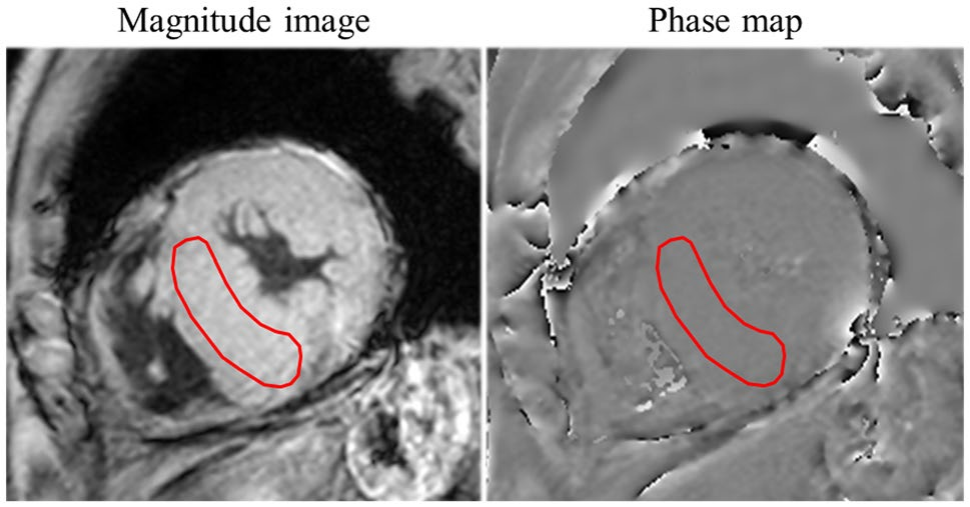
Measurement of myocardial susceptibility. Regions of interest (ROIs) were manually drawn on the septal midventricular wall on each phase map. The ROI on the magnitude image was copied and pasted onto the phase map. We calculated the average phase unit value for each echo time, and the phase shift slope as a quantitative parameter of myocardial susceptibility was obtained by the least squares method

For the quantification of myocardial susceptibility, regions of interest (ROIs) were manually drawn on the septal midventricular wall on each phase map by two cardiovascular radiologists with 10 and 13 years of experience in CMR. The ROI on the magnitude image was copied and pasted onto the phase map (Fig. 2). Myocardial ROI was drawn to include the segment from the epicardium to the endocardium with a 10–20% offset to avoid partial volume effects of the left ventricle or outer tissue of the epicardium. We calculated the average phase unit value using a software program (Image J, National Institutes of Health, Bethesda, MD, USA). The scale ranged from 0 to 4096, which is equivalent to − π to π radians. From the phase unit value for each echo time, the phase shift slope was obtained as a quantitative parameter of myocardial susceptibility based on the least squares method. A cardiovascular radiologist with 10 years of CMR experience interpreted and manually traced the LGE lesions and myocardium on LGE images. The calculated LGE volume was expressed as the % LGE of the myocardial volume of the left ventricle [% LGE = (LGE volume/myocardial volume) × 100].

### Statistical analysis

All numeric values were reported as mean ± standard deviation. Differences of the mean values between the CA and control groups with normally and non-normally distributed data were determined with the two-tailed independent *t*-test and Mann–Whitney *U*-test, respectively. The χ^2^ test was also used for comparisons between the two groups. One-way analysis of variance was used in multiple comparisons of the mean phase shift slope of patients with CA, controls, and healthy volunteers. If a significant difference was observed, then pairwise comparisons were performed with the Tukey’s test. The intraclass correlation coefficient (ICC) was calculated in order to determine the level of interobserver agreement for quantification of myocardial susceptibility. To identify significant parameters related to CA, univariate logistic regression analyses were performed, and the area under the receiver operating characteristic curve (AUC) of each parameter was calculated to assess the diagnostic accuracy. Results are presented in the form of odds ratio (ORs) with 95% confidence intervals (CIs) and *p*-values. Correlations were assessed with the Pearson correlation or Spearman coefficient. Differences of *p* < 0.05 were considered statistically significant. All statistical analyses were performed with the Bell Curve for Excel (Social Survey Research Information Co., Ltd., Tokyo, Japan).

## Results

### Baseline clinical characteristics

The baseline characteristics of our patients are presented in Table 1. There was no significant difference between the CA and control groups with respect to sex, age, height, weight, and body surface area. On echocardiography, the end-diastolic dimension was smaller, but the thickness of the interventricular septum and posterior wall of the left ventricle was greater in the CA group compared with that in the control group (*p* < 0.01). There was no significant difference in the end-systolic dimension and left ventricular ejection fraction between the groups. The ratio of early diastolic transmitral flow velocity to early diastolic mitral annular tissue velocity (*E*/*e*ʹ) was significantly greater in the CA group (*p* < 0.01). High-sensitivity cardiac troponin T (hs-cTnT) levels were significantly higher in patients with CA (*p* = 0.02).

**Table 1:** Patient characteristics and results

	CA group (*n* = 26)	Control group (*n* = 18)	*p*-value
Sex (male/female)	17/9	10/8	0.54
Age (years)	61.5 ± 13.9	59.0 ± 15.9	0.58
Body height (cm)	165.6 ± 9.2	161.8 ± 10.2	0.21
Body weight (kg)	57.6 ± 7.6	54.9 ± 12.6	0.39
Body surface area (m^2^)	1.63 ± 0.14	1.57 ± 0.21	0.26
eGFR (mL/min/1.73 m^2^)	61.3 ± 22.6	70.0 ± 16.8	0.17
BNP (pg/mL)	120.2 ± 120.5	67.3 ± 119.8	0.16
hs-cTnT (ng/mL)	0.035 ± 0.028	0.017 ± 0.020	0.02
QRS duration (ms)	116.4 ± 30.0	102.7 ± 19.2	0.10
LVDd (mm)	42.1 ± 5.6	46.2 ± 6.4	0.03
LVDs (mm)	28.2 ± 5.4	30.8 ± 6.5	0.16
IVSTd (mm)	13.8 ± 2.7	10.3 ± 1.6	< 0.01
PWTd (mm)	13.8 ± 3.1	9.9 ± 1.4	< 0.01
LVEF (%)	59.6 ± 10.4	56.6 ± 8.5	0.30
*E*/*e*′	17.2 ± 6.9	10.6 ± 5.7	< 0.01
*E*-deceleration time (ms)	226.3 ± 74.4	211.6 ± 46.5	0.46
Phase shift slope	0.25 ± 0.88	1.76 ± 1.15	< 0.01

Data are presented as mean ± standard deviations or actual values

*CA* cardiac amyloidosis; *eGFR* estimated glomerular filtration rate; *BNP* B-type natriuretic peptide; *hs-cTnT* high-sensitivity troponin T; *LVDd* left ventricular diastolic dimension; *LVDs* left ventricular systolic dimension; *IVSTd* intraventricular septal thickness in diastole; *PWTd* posterior wall thickness in diastole; *LVEF* left ventricular ejection fraction; *E* early diastolic transmitral flow velocity; *e*′ tissue Doppler-derived early diastolic peak velocity at the lateral mitral annulus

### Quantification of myocardial susceptibility

**Fig. 3:**
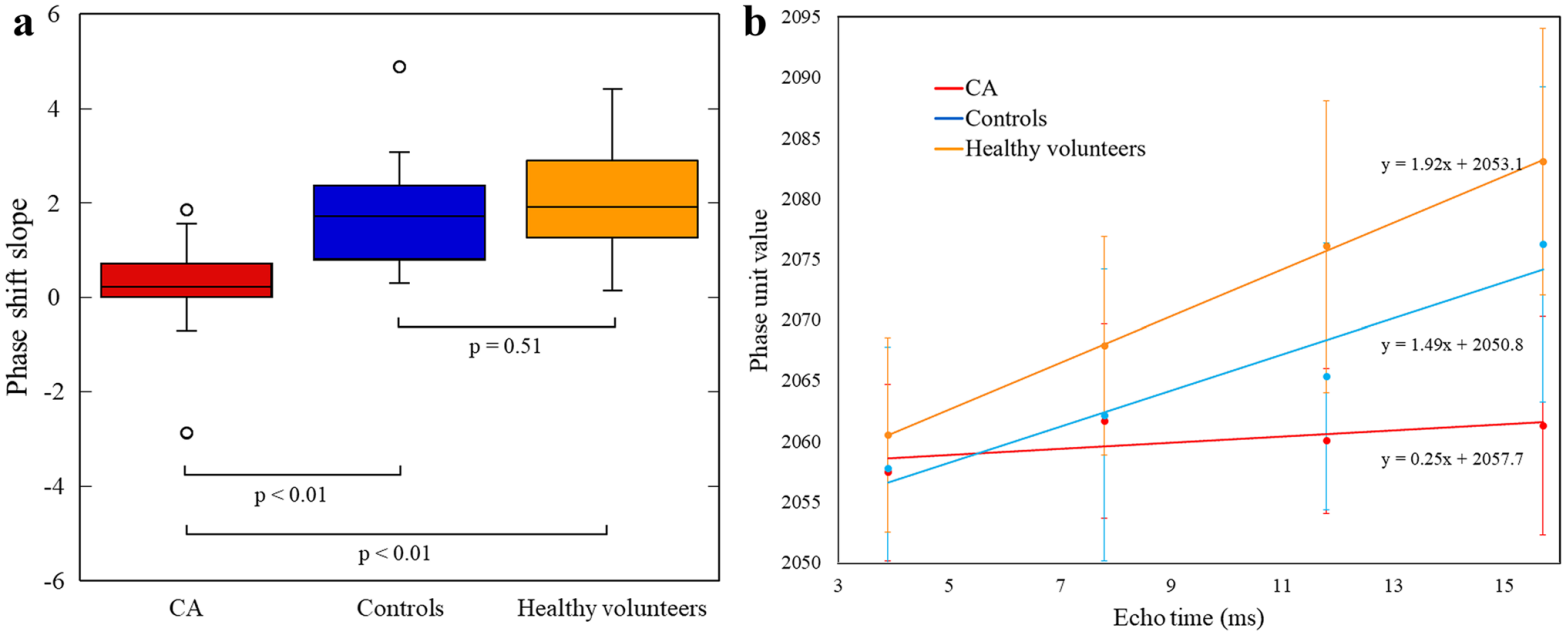
Quantification of myocardial susceptibility. **A** The phase shift slope, a quantitative parameter of myocardial susceptibility, was significantly lower in the cardiac amyloidosis (CA) group than in the control group and healthy volunteers (*p* < 0.01). There was no significant difference in the phase shift slope between the control group and healthy volunteers. **B** The graph shows the phase shift slope, a quantitative parameter of myocardial susceptibility, for the CA group, control group, and healthy volunteers. The phase shift slope was lower in the CA group compared with that in the control group and healthy volunteers

The phase shift slope, a quantitative parameter of myocardial susceptibility, was significantly lower in the CA group compared with that in the control group and healthy volunteers (*p* < 0.01) (Fig. 3). There was no significant difference in the phase shift slope between the control group and healthy volunteers. Excellent interobserver agreement was obtained for quantification of myocardial susceptibility with ICC of 0.934 (*p* < 0.01).

**Fig. 4:**
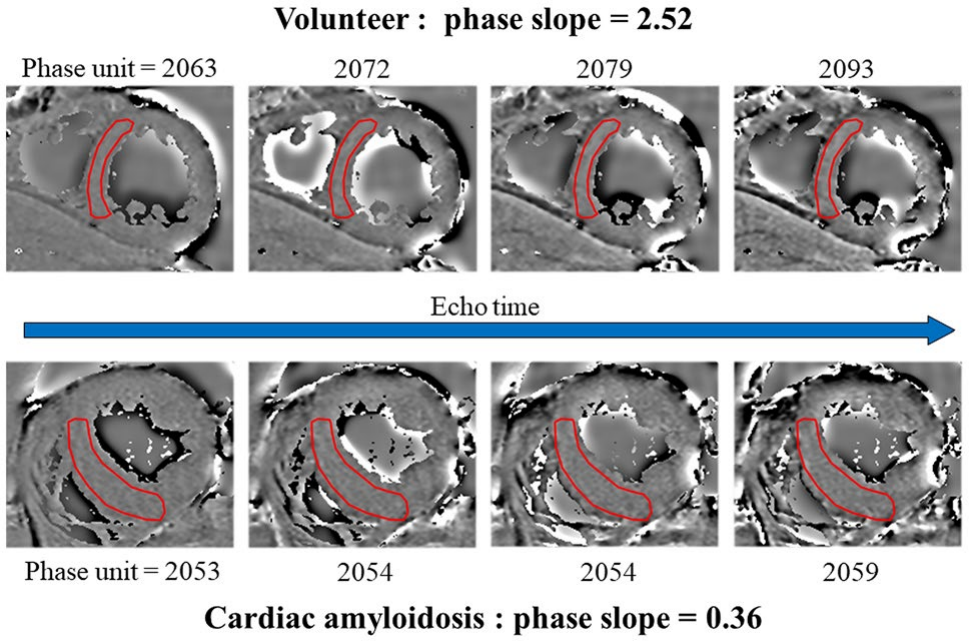
Myocardial susceptibility of CA patients and healthy volunteer. The phase shift and slope were greater in healthy volunteers than in patients with CA

Representative cases are shown in Fig. 4.

### Univariate logistic regression analysis of parameters related to CA

**Fig. 5:**
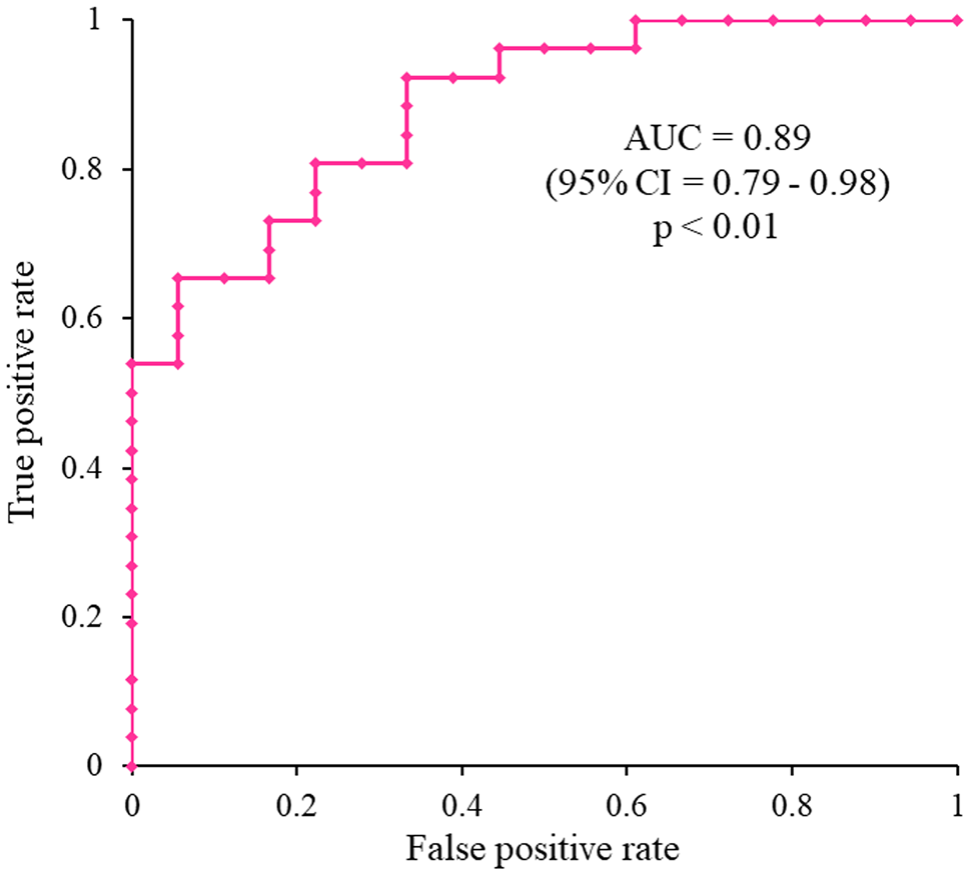
Receiver operating characteristic curve analysis for the prediction of CA. The phase shift slope yielded an area under the receiver operating characteristic curve of 0.89 (95% CI = 0.79–0.98) for the prediction of CA (*p* < 0.01)

Table 2 summarizes the results of the univariate analyses to identify predictors related to CA. From a total of 17 variables, 6 were considered significant predictors of CA (*p* < 0.05) during the univariate analysis, including IVSTd, PWTd, *E*/*e*ʹ, plasma B-type natriuretic peptide level, hs-cTnT level, and phase shift slope. The phase shift slope yielded the best AUC of 0.89 (95% CI = 0.79–0.98) for the prediction of CA (*p* < 0.01) (Fig. 5).

**Table 2:** Univariate logistic regression analysis for the prediction of CA

	OR	95% CI	*p*-value	AUC	95% CI
Sex (male/female)	2.361	0.689–8.092	0.172		
Age (years)	1.012	0.971–1.055	0.574		
Body height (cm)	1.044	0.977–1.114	0.203		
Body weight (kg)	1.029	0.965–1.098	0.380		
Body surface area (m^2^)	8.198	0.218–307.941	0.256		
eGFR (mL/min/1.73 m^2^)	0.977	0.945–1.010	0.175		
Ln BNP	5.366	1.407–20.462	0.014	0.723	0.556–0.889
Ln hs-cTnT	13.759	2.005–94.416	< 0.01	0.775	0.625–0.925
QRS duration (ms)	1.023	0.995–1.050	0.104		
LVDd (mm)	0.887	0.792–0.993	0.058		
LVDs (mm)	0.925	0.830–1.031	0.159		
IVSTd (mm)	2.113	1.384–3.226	< 0.01	0.852	0.747–0.957
PWTd (mm)	2.189	1.357–3.532	< 0.01	0.846	0.741–0.951
LVEF (%)	1.034	0.970–1.102	0.301		
*E*/*e*′	1.205	1.053–1.380	< 0.01	0.800	0.660–0.940
*E*-deceleration time (ms)	1.004	0.994–1.014	0.456		
Phase shift slope	0.121	0.034–0.423	< 0.01	0.890	0.794–0.983

*OR* odds ratio; *CI* confidence interval; *AUC* area under the receiver operating characteristic curve; *CA* cardiac amyloidosis; *eGFR* estimated glomerular filtration rate; *Ln* log-transformed; *BNP* B-type natriuretic peptide; *hs-cTnT* high-sensitivity troponin T; *LVDd* left ventricular diastolic dimension; *LVD* left ventricular systolic dimension; *IVSTd* intraventricular septal thickness in diastole; *PWTd* posterior wall thickness in diastole; *LVEF* left ventricular ejection fraction; *E* early diastolic transmitral flow velocity; *e*′ tissue Doppler-derived early diastolic peak velocity at the lateral mitral annulus

### Relationship between the clinical parameters and myocardial susceptibility in CA

As shown in Table 3, the phase shift slope was significantly correlated with the IVSTd (*r* =  − 0.39, *p* < 0.01) and PWTd (*r* =  − 0.35, *p* = 0.02) in all patients (controls and CA). No significant correlation between all clinical parameters and the phase shift slope was found in CA.

**Table 3:** Correlation between myocardial susceptibility (phase shift slope) and other clinical parameters

	All (controls and CA)		CA	
	*r*-value	*p*-value	*r*-value	*p*-value
Sex	0.06	0.70	0.30	0.12
Age	− 0.01	0.93	0.01	0.97
Body height	− 0.04	0.77	0.33	0.10
Body weight	0.01	0.97	0.31	0.10
Body surface area	− 0.02	0.91	0.33	0.10
eGFR	0.17	0.23	0.03	0.89
BNP	− 0.13	0.41	− 0.18	0.36
hs-cTnT	− 0.26	0.09	− 0.01	0.97
QRS duration	− 0.05	0.76	0.02	0.91
LVDd	0.21	0.17	0.20	0.31
LVDs	0.13	0.41	0.09	0.64
IVSTd	− 0.39	< 0.01	0.03	0.89
PWTd	− 0.35	0.02	0.05	0.79
LVEF	− 0.05	0.75	0.10	0.63
*E*/*e*′	− 0.23	0.14	− 0.08	0.71
*E*-deceleration time	− 0.05	0.76	− 0.01	0.97
% LGE	NA	NA	-0.20	0.31

*eGFR* estimated glomerular filtration rate; *BNP* B-type natriuretic peptide; *hs-cTnT* high-sensitivity troponin T; *LVDd* left ventricular diastolic dimension; *LVDs* left ventricular systolic dimension; *IVSTd* intraventricular septal thickness in diastole; *PWTd* posterior wall thickness in diastole; *LVEF* left ventricular ejection fraction; *E* early diastolic transmitral flow velocity; *e′* tissue Doppler-derived early diastolic peak velocity at the lateral mitral annulus; *LGE* late gadolinium enhancement; *NA* not available

## Discussion

We demonstrated that the phase shift slope that serves as a quantitative parameter of myocardial susceptibility was significantly lower in the CA group than in the control group and healthy volunteers and identified it as a predictor of CA. The predictive ability of the myocardial susceptibility was high, with an AUC of 0.89. The myocardial susceptibility was significantly correlated with the IVSTd and PWTd in all patients. This observation may be of practical importance and identifies myocardial susceptibility analysis by unenhanced CMR as a noninvasive technique for quantitative marker of patients with CA. Although the underlying etiology for the change in myocardial susceptibility in CA has been unknown, possible causes include the iron distribution, metabolic oxygen consumption, blood degradation, calcification, myocardial anisotropy, and other pathophysiological conditions.

Verifying the myocardial susceptibility and its underlying mechanisms using CMR may lead to improved techniques for the examination of microstructure of the heart. Myocardial fiber organization and structure are important determinants of myocardial stress and strain. These are altered by cardiac hypertrophy, fibrosis, infarction, and material deposition. They also seemed to play an important role in arrhythmogenesis. In CA, extracellular ß-pleated amyloid deposits cause disruption in myocardial architecture [10]. Hence, assessing myocardial anisotropy by CMR susceptibility analysis may potentially be used to quantify the functional properties of healthy and diseased hearts.

Changes in myocardial susceptibility do not appear to be disease specific, but this needs further validation. According to our results, myocardial susceptibility analysis can be used in the assessment of cardiac involvement in patients with systemic amyloidosis or in cases wherein CA is highly suspected. Moreover, myocardial susceptibility analysis by CMR has the potential to serve as a noninvasive imaging marker for disease surveillance, possibly contributing to the management of patients with CA.

T2*-weighted GRE CMR has shown potential as a method for the assessment of myocardial susceptibility [11]. The GRE image phase is sensitive to changes in the magnetic field caused by magnetically susceptible components in tissues, such as deoxyhemoglobin, deoxymyoglobin, and calcification, and can be used in the determination of susceptibility differences among tissues. In fact, T2* quantification is currently the method of choice for myocardial tissue iron assessment [12]. The myocardial susceptibility analysis in this study may reflect subtle magnetic susceptibility changes due to other substances and iron.

Myocardial anisotropy is assessed with diffusion tensor imaging and histology [13–15]. Even though diffusion tensor imaging is a nondestructive imaging modality, it is challenged by spatial resolution limits and long scan times imposed by low signal-to-noise ratios. Conversely, histological techniques can provide whole-heart myocardial anisotropy assessment with extremely high-spatial resolution but are labor-intensive and require organ harvesting. Considering the high-resolution capability of GRE phase imaging and recent developments in susceptibility tensor imaging using CMR, the imaging of the myocardial susceptibility anisotropy may aid in the assessment of myocardial fiber integrity and alterations induced by cardiac diseases and disorders [16].

Myocardial T1 mapping that is typically used to evaluate the myocardial T1 relaxation time facilitates noninvasive detection and quantification of biologically important processes associated with myocardial edema, fibrosis, and material deposition. Unenhanced T1 mapping or native T1 mapping reflects myocardial diseases, involving the myocyte and interstitium, without use of gadolinium-based contrast agents. Furthermore, contrast-enhanced T1 mapping enables the calculation of the extracellular volume (ECV) fraction. CA is associated with significantly elevated native T1 and ECV values relative to those documented in other cardiac diseases, demonstrating the high-diagnostic precision of T1 mapping in CA detection [5, 17]. Based on the previous research reports, myocardial susceptibility analysis is unlikely to have superiority over myocardial T1 mapping in the diagnostic ability of CA. However, myocardial susceptibility analysis reflects a different disease mechanism to myocardial T1 mapping. Therefore, the clinical significance of myocardial susceptibility analysis in CA is currently unknown. We believe that the myocardial susceptibility used can be one of the features that can be used for multiparametric analysis along with the T1 mapping. Further research is warranted to determine the utility of myocardial susceptibility analysis in CA.

Recently, Wen et al. investigated the feasibility of quantification of myocardial susceptibility using CMR, and reported that mixed-venous oxygen saturation which is an important indicator of cardiac function can be measured non-invasively [18]. Deoxyhemoglobin increases blood magnetic susceptibility, and that can be measured using this CMR technique. The same research group also reported that the quantification of myocardial susceptibility could find a difference in blood oxygenation between the left and right ventricles which is a key index of cardiac performance [19]. In our study as well, differences in intramyocardial oxygenation between CA and controls may have influenced the results. The quantification of myocardial susceptibility by CMR, unlike other quantitative mappings, may reflect a multifaceted state in the myocardium.

This study had several limitations. First, the number of patients was small, and the study was performed at a single center. Validation with a similar cohort would be useful to support our results, but this cohort was unavailable. This is the first cohort study in which patients with CA underwent CMR myocardial susceptibility analysis that constitutes the novel and creative features of our study. Large-scale prospective clinical studies are required for rigorous evaluation of the feasibility of our methods. Second, we did not examine the relationship between histological findings and myocardial susceptibility results. Third, we did not evaluate the myocardial susceptibility for cardiac diseases other than CA, such as hypertrophic cardiomyopathy, dilated cardiomyopathy, hypertensive heart disease, etc. The applicability of CMR myocardial susceptibility analysis for the characterization of these cardiac diseases should be explored. Lastly, we did not compare the myocardial susceptibility findings in CA subtypes because the sample size was too small for meaningful comparative analyses. Large-scale comparative studies are needed to define the myocardial susceptibility features of each type of CA.

In conclusion, myocardial susceptibility analysis by unenhanced CMR helps the diagnosis of patients with CA. The quantification of myocardial susceptibility by CMR may offer new insights for cardiac diseases and can be a new quantitative imaging biomarker for CA. Additional pathological studies are needed to explain the relationship between the observed phenomenon and underlying causes.
